# Responsibility of Writers

**Published:** 1878-07

**Authors:** 


### RESPONSIBILITY OF WRITERS.

THE man dressed in spotless white will not fail to have his garments blackened, if he mingles among a crowd of sweeps. There are clergymen who cannot feel authorized to occupy the pulpit of persons claiming to be clergymen too, for fear it should be construed to countenance the supposed errors of the latter. No man of position can allow himself to associate, without prejudice, with the profane, the Sabbath-breaker, the drunken, and the licentious, for he lowers himself, without elevating them. The sweep is not made the less black by rubbing against the well-dressed and the clean, while they are inevitably defiled.

If a good man buys a bad book, or writes a commendatory preface to a bad book, he gives both his countenance. If he writes for a bad publication, he, in a measure, endorses its sentiments. To write an article, be it ever so good, for a periodical, each number of which is, in the main, filled with third-rate fictions, or even first-rate fictions, is to endorse that publication in the main. If a good man writes for such, in the hope of slipping in a wholesome truth now and then, where it would not be otherwise done at all, it is as if he coated a poisonous pill with sugar, or mingled a serpent's venom with honey; the poison and the venom are too predominating; they still destroy, while the sweetness is all lost.

If able men, for a dollar or two a page, or column, will write for flash newspapers and flash magazines, which, without their fictions of words, and falsehoods of pictures, would not sell at all, they simply aid to bolster up a lie, and pander to the credulities of an ignorant public. To palm off the picture of an artist's brain for that of an actual occurrence, to give the portraits of the *passé* and the dead for those of living criminals, is a falsehood and a cheat, as much as the publication of a fiction for an actual fact

